# Effect of Corrosion Protection Method on Properties of RSW and RFSSW Lap Joints Applied in Production of Thin-Walled Aerostructures

**DOI:** 10.3390/ma13081841

**Published:** 2020-04-14

**Authors:** Agata Dudek, Jacek Andres, Agata Wrońska, Waldemar Łogin

**Affiliations:** 1 Department of Production Engineering and Materials Technology, Czestochowa University of Technology, 42-218 Czestochowa, Poland; dudek@wip.pcz.pl; 2 Polskie Zakłady Lotnicze Sp. z o.o.A Lockheed Martin Company, 39-300 Mielec, Poland; agata.wronska@lmco.com (A.W.); waldemar.login@lmco.com (W.Ł.)

**Keywords:** resistance spot welding, refill friction stir spot welding, alcladed aluminum metal sheets, sealant, anodic layer

## Abstract

Aluminum structures, and in particular an element of aerostructures, are strongly exposed to the effects of weather conditions. In the case of using new techniques of joining these structural elements, the selection of proper corrosion protection without losing the required properties of the joint may determine its potential application. This paper presents the results of experimental research concerning the influence of corrosion protection on the microstructure and mechanical strength of resistance spot welded (RSW) and refill friction stir spot welded (RFSSW) joints. The tests were carried out on 2024 T3 aluminum alloy, both sides alcladed. For comparison purposes, the following joints were welded: without any protection, with the primer layer, with anodic oxide coating, and with anodic oxide coating plus sealant between the overlapping surface of the welded metal sheet. The samples were visually inspected, and metallographic and mechanical strength examination was conducted. The test results indicate that the application of the protective layers and its type have an impact on the strength of RSW and RFSSW joints. The use of an adhesive or sealant in welded joints provides an increase in the load capacity of the joint.

## 1. Introduction

Aluminum alloys are the basic group of metallic construction materials used to build aircraft structures. They are gradually displaced by more and more advanced composite materials. However, their usage is still wide, and is related to easy manufacture, non-toxicity, relatively good strength, and corrosion resistance in industrial and marine environments [1]. Aluminum grades that are the most commonly used in aviation include the 2xxx (Al alloys in which copper is the principal alloying element) and 7xxx (aluminum–zinc–magnesium alloys) series. This study focuses on the characteristics of various types of joints of elements made of 2024 T3 alloy. This material is characterized by good strength, high yield strength, and formability, which is particularly important in the case of aircraft construction. The performance of this alloy is mainly the result of the chemical composition and morphology of microstructure phase components, which are directly affected by heat treatment. The basic alloy addition in this group of materials is copper, which plays a key role in the strengthening process, but also has an adverse effect on corrosion resistance [2,3]. Chemical composition of the 2024 alloy is shown in Table 1. The 2xxx series of Al alloys are susceptible to general, pitting, transcrystalline, intergranular, stress, and exfoliation corrosion. Resistance to stress corrosion or various forms of intergranular corrosion can be improved by appropriate heat treatment, but this does not affect general and pitting corrosion. Therefore, these alloys, including 2024 T3, are protected by a layer of alclad, e.g., a layer of alloy 1xxx (group includes Al alloys of 99.0% or higher purity of aluminum) series, which ensures resistance to pitting corrosion and does not worsen strength properties [2]. Supplied sheets were double-sided, covered with alclad 1230 alloy. The average alclad thickness was equal to 4% of total sheet thickness per each side. Chemical composition of alclad 1230 is given in Table 1.

During aerostructure construction, particular attention should be paid to the anti-corrosion protection of the contact surface of the joined elements, e.g., skin with stringers, which should be protected against the penetration of environmental factors. Protection methods that are currently used in the construction of airframes in PZL Mielec, such as resistance spot welded (RSW) joining on liquid primer with epoxy adhesive application, are less effective.

In aviation, additional forms of structural element surface protection are often used: anodizing (formation of the aluminum oxide layer in electrolytic processes), chromate coatings, sol-gel coatings and others, primers, and in the case of lap joints protection, various types of adhesives and sealants [2,4].

The commonly used conversion coating produced by the anodizing process offers very good protection against electrochemical corrosion. Unfortunately, this type of protection is not applicable in the case of RSW welding, both before and after welding. The refill friction stir spot welded (RFSSW) process, which is possible in the case of anodized sheets welding, is much more promising. Replacement of RSW technology with RFSSW would make it possible to improve the corrosion resistance of structural elements made of aluminum alloys.

Another important feature of the Al 2xxx series alloys, which is considered in the design of the structure, is poor weldability. Therefore, critical elements of aviation structures, such as skin and its stringer reinforcements, are usually connected mainly by riveting and by means of resistance spot welding as the main connector-free methods. Welding techniques are rather excluded because in these type of alloys (2024 T3 is particularly susceptible), there is a high risk of the occurrence of non-acceptable defects within the joint and heat-affected zone (HAZ): porosity, hot cracking, including also liquid cracking. It should also be emphasized that aluminum alloys have a high affinity for oxygen, which results in the formation of refractory Al_2_O_3_ oxide on their surface, and the secondary reasons are high thermal conductivity and high thermal expansion, which usually lead to significant deformations of the welded structure [5,6,7].

Resistance spot welding (RSW) is especially recommended for joining elements made of Al 2024 alloy, because it is characterized by high repeatability, ensures a smooth surface at the joining area, and has relatively good mechanical strength. The RSW welding process is easy to automate, hence it is widely used in mass production, mainly of steel structures in the automotive industry. In the case of resistance welding of aluminum alloys, special requirements must be provided. These metals are characterized by high electrical and thermal conductivity, and lower electrical resistance in relation to steel, which determines the necessity of using a high welding current (2 to 3 times higher than in the case of steel). The consequence of this is high energy consumption during welding. In addition, this process is particularly sensitive to the surface quality of joined elements, hence for aluminum alloys, etching (removal of non-conductive oxides) and ensuring high purity of the welded elements’ surface are required. Oxides cause a significant increase in contact resistance and the amount of supplied heat, and as a result, rapid consumption of electrodes [8,9]. Inappropriate welding parameters of RSW may cause the following defects in spot welds: too small a diameter of the weld, lack of penetration, inadequate geometry of the weld, porosity and cracks, and too deep plunging of electrodes in the joined materials [10].

An alternative joining method for conventional joining techniques is refilled friction stir spot welding (RFSSW). This process is carried out without the use of a liquid phase, below the melting point of the material, hence there is no risk of defects typical for welded joints. Welding is carried out by using a special tool built from three elements: a spindle, an inner sleeve, and a clamp. The principle of operation and the mechanism of weld formation are described in detail in many scientific papers, e.g., [11,12,13]. The basic advantages of this technology include good surface quality of welds, small deformations of joined elements, low material losses, low energy consumption, no emission of pollutants, as well as ease of automation, and above all, no need for special preparation of joined parts. If the basic parameters of the RFSSW process: depth of the inner sleeve cavity, weld time, rotational speed of the mandrel and pin are correct, it is possible to obtain welds free from typical disadvantages: poor material mixing, voids, hooking, or excessive penetration of the tool [14].

In this work, the RSW and RFSSW joints of Al 2024 sheets of metal were characterized and compared. The authors focused on the microstructure and mechanical strength of lap joints in relation to different surface conditions of joined elements and various anti-corrosion protections on the overlapping surfaces.

## 2. Materials and Methods

The research concerned the use of Al 2024 T3 alloy sheets (chemical composition according to AMS-QQ-A-250/5), coated on both sides with a layer of alclad with a thickness corresponding to 4% of the detail thickness.

**Figure 1:**
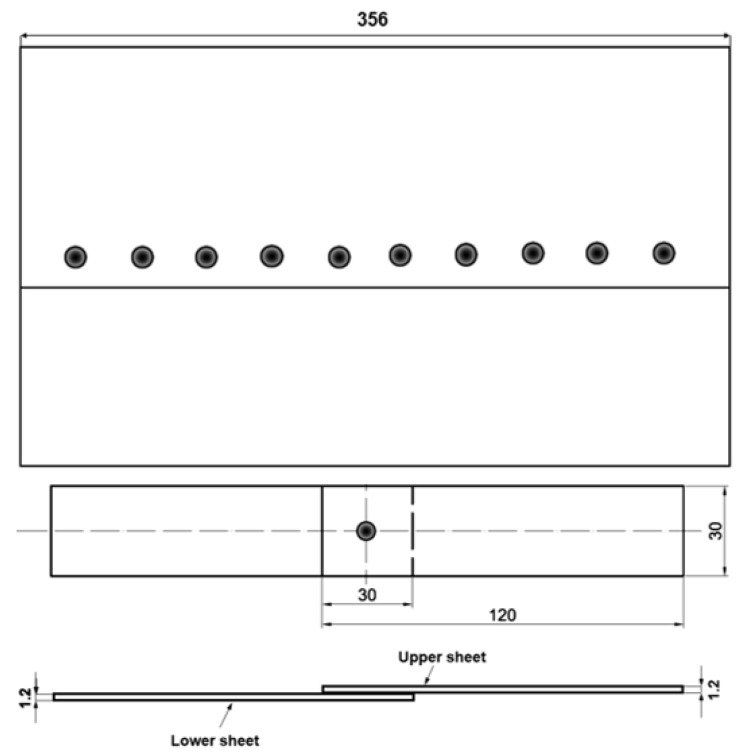
Scheme of sheets and test specimen welded in lap configuration.

Metal sheets of the following dimensions, 356 mm × 10 mm and a thickness of 1.2 mm (top and bottom plate) were joined. The sheets were welded in lap configuration in the rolling direction. Test samples were cut out from the sets of spot joints: 8 samples for mechanical strength test and two for optical microscopy and microhardness measurements. All welded joints were visually inspected. The scheme of the test specimen with a single spot joint and with characteristic dimensions is shown in Figure 1.

The joints were made using two welding technologies: RSW and RFSSW. The surface of the sheets was prepared using several different ways of protecting the material from corrosion. One set of test RSW and RFSSW samples was made without surface protection and for this purpose, the surface of the sheets was etched (RSW joint) or degreased (RFSSW joints). In the RSW process, degreasing was not sufficient because all oxides should also be removed to maintain the same resistivity on all welded surfaces. In the case of RSW joints, the area of overlaps was coated with a layer of liquid primer before welding and epoxy adhesive after welding. For RFSSW joints, plates with a degreased surface, anodic oxide coating was used, and the layer of sealant was distributed on the lap surfaces. Two types of substances were used for sealing. The RSW welding process was carried out on a Soudronic resistance-type PM3b welding machine (Soudronic Holding AG, Bergdietikon, Switzerland) while the RFSSW welding process was carried out on an RPS-100 welding machine (Harms and Wende, GmbH & Co. KG, Hamburg, Germany). Both types of joints were made for the same sets of parameters, selected on the basis of preliminary tests [15].

The exclusion of the influence of welding parameters on the properties of joints made it easier to evaluate the impact of applied corrosion protection on the microstructure and strength of joints.

The metallographic samples were cut along the axial direction, and then mounted in resin, grinded with abrasive paper of different gradations, and polished using aluminum oxide aqueous suspension. Next, the samples were etched at ambient temperature in Keller’s reagent (5 mL HF, 15 mL HCl, 25 mL HNO_3_, 955 mL H_2_O). Finally, etching for 20–30 s was done until the microstructure was revealed. The prepared transverse specimens were subjected to microstructural observations on the Zeiss digital optical microscope.

**Figure 2:**
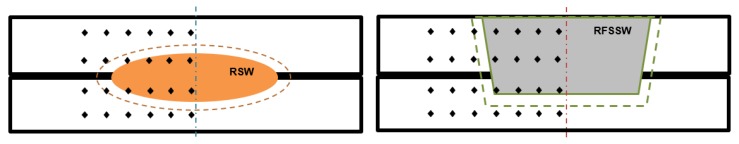
Scheme of microhardness indentations distribution in resistance spot welded (RSW) and refill friction stir spot welded (RFSSW) cross-section.

Microhardness measurements were made on transverse cross-sections on the Buehler Wilson VH3100 automatic microhardness tester (Buehler Ltd., Lake Bluff, IL, USA) at 50 G load. A total of 20 and 28 indentations were respectively taken for the RSW and RFSSW samples. All measurements were carried out at least three days after welding to maintain sufficient time for the natural aging process in all samples. The cross-section of the weld in both cases was symmetric. Hence the measurement indentations matrix included only the area from the heat-affected zone (HAZ) to the weld nugget in accordance with the diagrams in Figure 2. The hardness of the parent material was measured a few millimeters from the weld at the same load value. The mean microhardness value was equal to 136 HV0.05 (based on 10 indentations).

Tensile strength tests of joints were carried out on the MTS Landmark machine, equipped with force and displacement sensors. A breaking force (kN) was recorded for each tested sample. The broken samples were visually inspected to determine the fracture mechanism.

## 3. Results and Discussion

**Figure 3:**
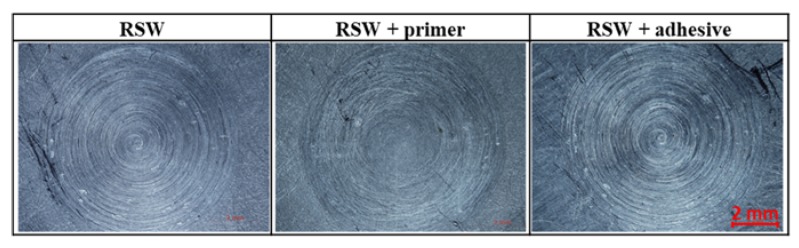
RSW weld face of examined samples.

**Figure 4:**
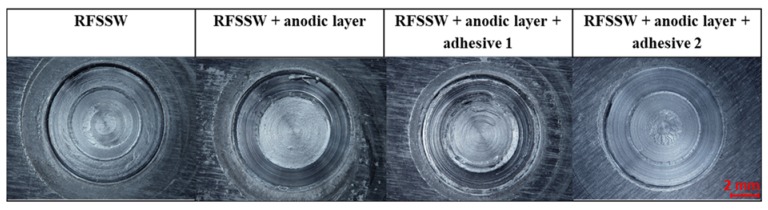
RFSSW weld face of examined samples.

The sets of spot welded joints of 2024 T3 sheets with various types of anti-corrosion protection were subjected to visual observations. RSW welds were characterized by an acceptable face and lack of discontinuities (Figure 3). However, on the edge of the RFSSW welds face, material loss was noticed, which indicates that the hole after the retreating sleeve was not fully filled after the tool sleeve came out. (Figure 4). The presence of this defect was evaluated during microscopic examination of individual test sample cross-sections.

**Figure 5:**
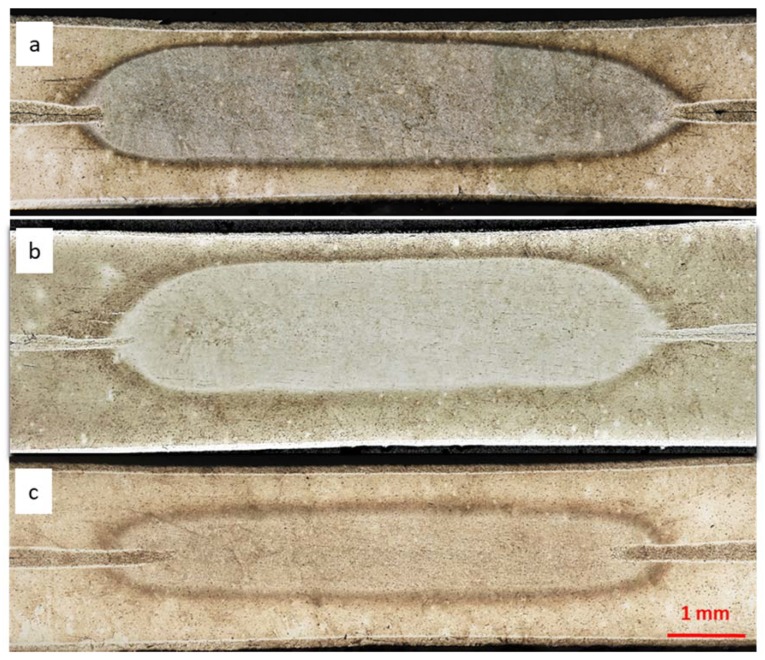
The microstructure of resistance spot welds made on the plates, with different surface preparation: (**a**) etching before welding, (**b**) etching and primer coating before welding, (**c**) etching before welding, and application of adhesive in the area of the overlap after welding.

**Figure 6:**
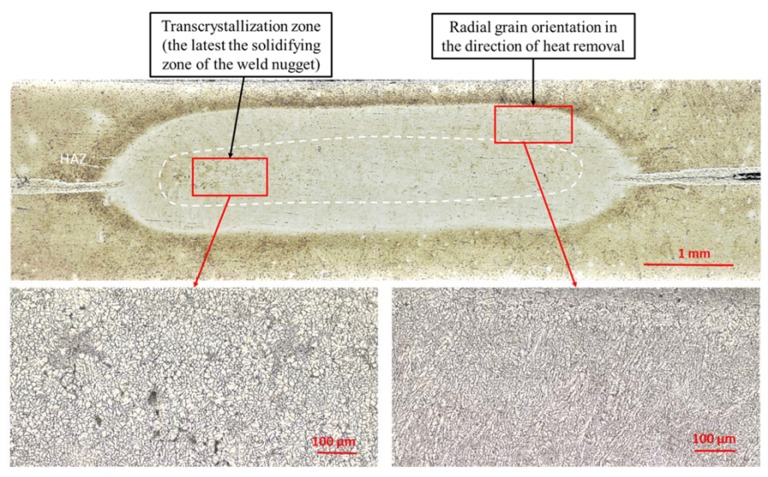
Weld nugget zones in RSW joint.

**Figure 7:**
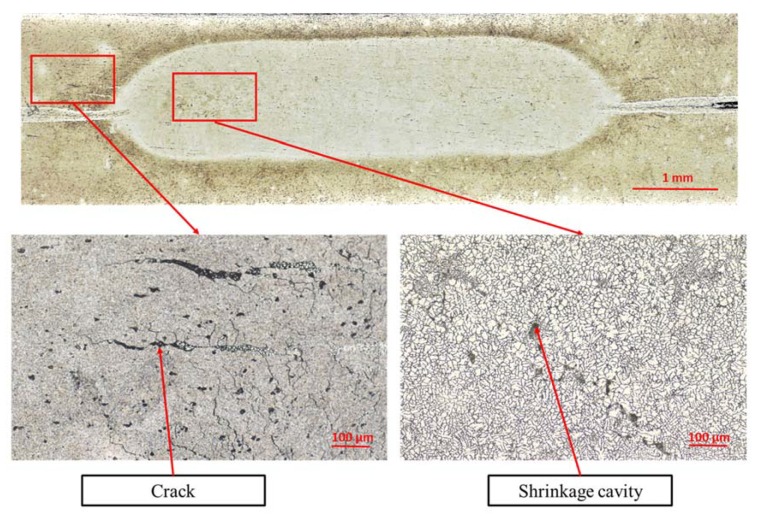
Microstructural discrepancies in RSW joints.

The RSW weld nugget coexisted in two characteristic areas (Figure 5, Figure 6 and Figure 7): outer, in which the grains were oriented radially, in the direction of heat removal during the crystallization of remelted material. In the central part of the welding core, there was a transcrystallization zone, solidifying at the latest; its microstructure was built of very fine equiaxed grains. According to [16], in the transcrystallization zone, contamination in the form of metallic and non-metallic inclusions and defects, such as cracks and voids, can often occur, which makes this zone the weakest point of the weld.

In the case of RSW welds, microscopic observations revealed the presence of dispersed micropores in the area of transcrystallization and cracks in the heat-affected zone (Figure 7). These defects are often observed in the RSW joints of Al alloys and are the result of material shrinkage during solidification of the remelted material, high heat dissipation coefficient, and high cooling rate. However, it should be emphasized that the presence of small cracks or fine pores have a minor effect on the mechanical (both static and fatigue) strength of RSW joints. Mechanical properties are mainly dependent on nugget size, which means that electrode force, welding time, and welding current are the key factors [17].

**Figure 8:**
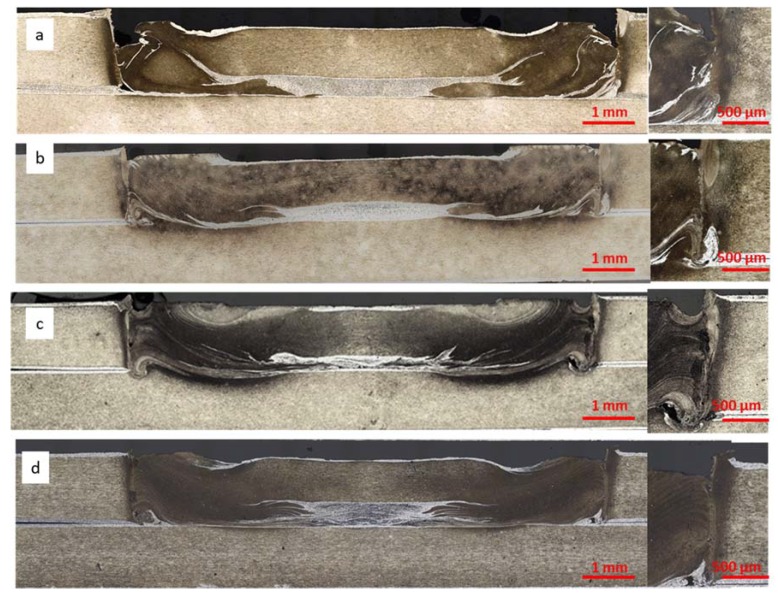
The microstructure of RFSSW made on the plates, with different surface preparation: (**a**) degreasing before welding, (**b**) anodizing before welding, (**c**) anodizing and application of sealant 1 on the overlap surface before welding, (**d**) anodizing and application of sealant 2 on the overlap surface before welding.

In this paper, the authors focused among others on RFSSW joint quality when there was an applied additional protective medium on the surface of the joined elements, using nomenclature from work [15], where particular zones and internal defects occurring in RFSSW joints of alcladed aluminum sheets were described. Investigations of the RFSSW microstructures confirmed the occurrence of material losses at the interface of the sleeve and thermomechanical deformation zone, which were previously observed during visual tests on the welds face in the form of craters. The largest loss of material was found in the joint of alcladed metal sheets, without an additional protective layer (Figure 8a). This is due to the fact that the alclad on the surface of the sheets, which is more plastic than the substrate, sticks to the surface of the tool and makes it difficult to refill sufficiently the spot weld after tool retreating.

In addition, the area of the accumulated plater was observed in the central part of the RFSSW welds, also referred to in literature as the bonding ligament. Because this area is in the shear plane of the joint, it can weaken the joint and promote shear damage. In the case of samples welded on sealant 1 and 2 (Figure 8c,d), poor material mixing was observed at the interface of the inner sleeve mixing area. On all samples, voids were found mainly in the lower part of RFSSW welds in the impact area of the tool sleeve and the welded samples with sealants 1 and 2 also on the boundary of the thermomechanical deformation zone and weld nugget (Figure 8c,d).

**Figure 9:**
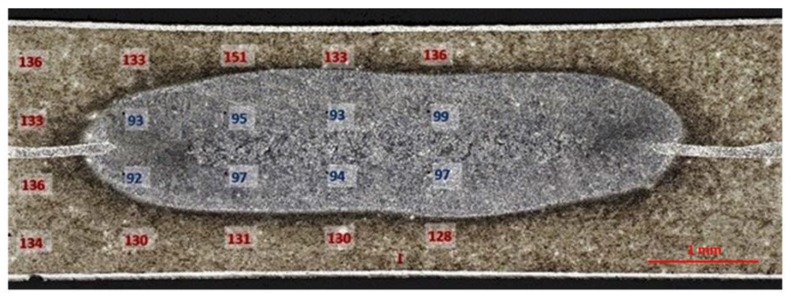
Microhardness of the resistance spot weld.

**Figure 10:**
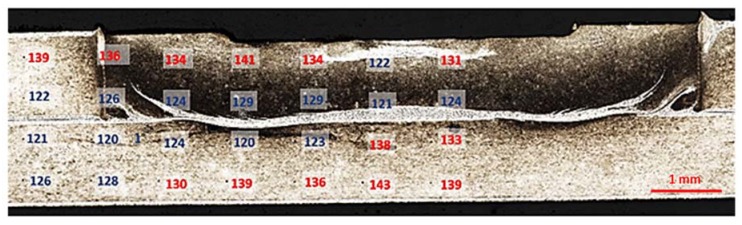
Microhardness of RFSSW.

In the case of RSW weld, microhardness measurements showed a significant decrease in hardness of the weld nugget zone (Figure 9) of about 40 HV0.05 in comparison to the hardness of HAZ and the parent material. This is a characteristic feature of RSW joints of precipitation-strengthened alloys: the decrease in hardness results from the reversing of the strengthening effects that occur because of remelting and material crystallization in the core of the weld nugget zone. In RFSSW joints, differences in hardness between the weld area and other joint zones were significantly lower (Figure 10). In the area of material mixing as well as in HAZ and the thermomechanical deformation zone, the hardness was in the range of 120–130 HV0.05. The scatter of results was small despite significant microstructural differences. The highest hardness values (130–143 HV0.05) were recorded in the area just below the surface of the weld, in HAZ, the thermomechanical-affected zone, and in the bottom sheet below the mixing zone. These areas remained in contact with the tool elements (from above) and anvil (from the bottom) for the longest time. However, to determine the cause of the increased hardness in these areas, it is necessary to carry out a detailed microstructural analysis and identify the morphology of the precipitates. This will allow evaluation of the influence of pressure of the tool and anvil and the heat generated by these elements on microstructural changes and microhardness. The issue will be continued in future studies.

**Figure 11:**
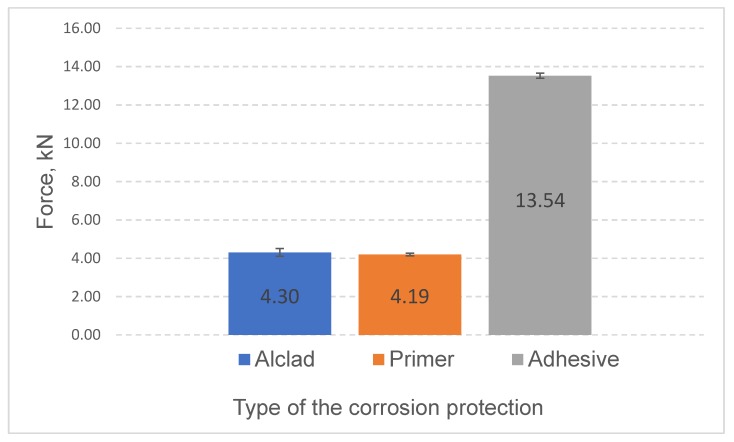
Static strength test results for RSW joints.

**Figure 12:**
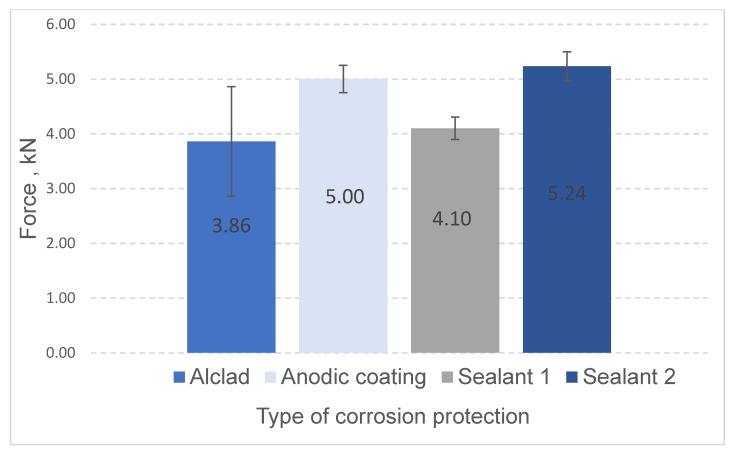
Static strength test results for RFSSW joints.

Static strength test results are shown in Figure 11 and Figure 12. RSW joining with liquid primer does not cause significant changes in joint strength; the value of the breaking force is comparable with the strength of the joint of the aluminum sheets without any protection (Figure 11). On the other hand, hybrid joints, protected by application of epoxy adhesive on the contact surface of the overlap after welding, were characterized by over three times higher strength. Much higher mechanical strength results from the fact that in the hybrid joints, the load-bearing surface is much larger than the surface of the RSW weld itself.

In the case of RFSSW joints, the highest load capacity was demonstrated by the welded joints with sealant 2. In contrast, the lowest load capacity was demonstrated by the joints of metal sheets without additional surface preparation (only with alclad layer on the surface) (Figure 12). Additional protection of the anodic oxide coating, the hardness of which was higher than the substrate material and alclad layer, promoted more effective mixing of the weld material, and reduced the risk of creating a crater around the weld, because welded material did not adhere to the tool’s working surface. This resulted in higher load capacity (5 kN) than in the case of alcladed plates without an anodized layer. The highest strength (5.24 kN) was registered for welded joints with sealant 1. In the case of welded joints with sealant 2, the strength was lower (4.10 kN). The reason for this phenomenon may be the lower resistance of sealant 1 to the high temperature (~350 °C in the mixing zone) generated during the welding process, lowering its shear strength.

**Figure 13:**
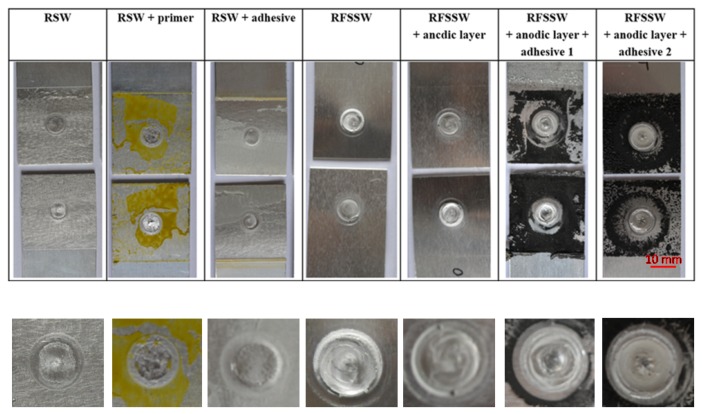
The surface morphologies of RSW and RFSSW joints after breaking.

Figure 13 shows selected test samples with different protections after strength tests. All types of samples during strength tests were broken mainly by shearing the weld along the contact line of both sheets. The destruction of the RSW joint in the middle of the weld by separation of the upper and lower plates was common and was caused by a structural notch. In contrast, the destruction of RFSSW joints also occurred at the contact of two sheets but as a result of alclad concentration in the weld. It had poor strength compared to base material (2024 T3) and also created a notch susceptible for fracturing. The adhesive used in the RSW joint and the sealants applied in the RFSSW joints were characterized by good adhesion to the surface of the lap joints. Small losses of sealant 1 and 2 that were visible around the RFSSW weld (Figure 8) were created as a result of their squeezing out from this area under the tool pressure in the initial stage of the welding process.

## 4. Conclusions

The test results have led to the following conclusions:

- Application of liquid primer before the RSW process does not affect the static strength (4.19 kN) of the tested joints (in comparison to the joints without primer, −4.30 kN).
- Application of epoxy adhesive causes a significant, more than three times, increase of static strength (13.52 kN) of the RSW joints.
- By using RFSSW technology, it is possible to join elements protected with an anodic oxide coating and elements with a sealant layer applied on overlap surfaces, which is impossible in the case of RSW welding.
- Application of proper sealant does not interfere with the RFSSW joining process, slightly increases the load capacity (5.24 kN) of RFSSW joints of anodized sheets (5.00 kN), and may protect the overlap area from an aggressive environment.

## Figures and Tables

**Table 1:** Chemical composition of 2024 alloy and alclad 1230.

Element	2024	Alclad 1230
	Mass %	
Cu	3.8–4.9	max 0.10
Mg	1.2–1.8	max 0.05
Mn	0.3–0.9	max 0.0
Fe	max 0.5	*
Si	max 0.5	*
Cr	max 0.1	-
Zn	max 0.25	max 0.10
Ti	max 0.16	max 0.03
Others, separately	max 0.05	0.03
Others, total	max 0.15	-
Al	Balance	min 99.30

* Maximum 0.7% iron plus silicon.
